# Evaluation of soybean genotypes for reaction to natural field infection by *Cercospora* species causing purple seed stain

**DOI:** 10.1371/journal.pone.0222673

**Published:** 2019-10-10

**Authors:** Shuxian Li, Gabe Sciumbato, Debbie Boykin, Grover Shannon, Pengyin Chen

**Affiliations:** 1 United States Department of Agriculture, Agricultural Research Service (USDA, ARS), Crop Genetics Research Unit, Stoneville, Mississippi, United States of America; 2 Mississippi State University, Delta Research and Extension Center, Stoneville, Mississippi, United States of America; 3 USDA, ARS, Stoneville, Mississippi, United States of America; 4 Division of Plant Sciences, University of Missouri, Portageville, Missouri, United States of America; University of Guelph, CANADA

## Abstract

Purple seed stain (PSS) of soybean (*Glycine max* (L.) Merr.) is a prevalent seed disease. It results in poor seed quality and reduced seed lot market grade, and thus undermines value of soybean worldwide. The objectives of this research were to evaluate the reaction of selected soybean genotypes collected from 15 countries representing maturity groups (MGs) III, IV, and V to PSS, and to identify new sources of resistance to PSS based on three years of evaluation of natural field infection by *Cercospora* spp. in the Mississippi Delta of the U. S. In this study, 42 soybean genotypes were evaluated in 2010, 2011, and 2012. Seventeen lines including six MG III (PI 88490, PI 504488, PI 417361, PI 548298, PI 437482, and PI 578486), seven MG IV (PI 404173, PI 346308, PI 355070, PI 416779, PI 80479, PI 346307, and PI 264555), and four MG V (PI 417567, PI 417420, PI 381659, and PI 407749) genotypes had significantly lower percent seed infection by *Cercospora* spp. than the susceptible checks and other genotypes evaluated (*P ≤* 0.05). These genotypes of soybean can be used in developing soybean cultivars or germplasm lines with resistance to PSS and for genetic mapping of PSS resistance genes. In addition, among these 17 lines with different levels of resistance to PSS, nine soybean genotypes (PI 417361, PI 504488, PI 88490, PI 346308, PI 416779, PI 417567, PI 381659, PI 417567, and PI 407749) were previously reported as resistant to Phomopsis seed decay. Therefore, they could be useful in breeding programs to develop soybean cultivars with improved resistance to both seed diseases.

## Introduction

**Fig 1:**
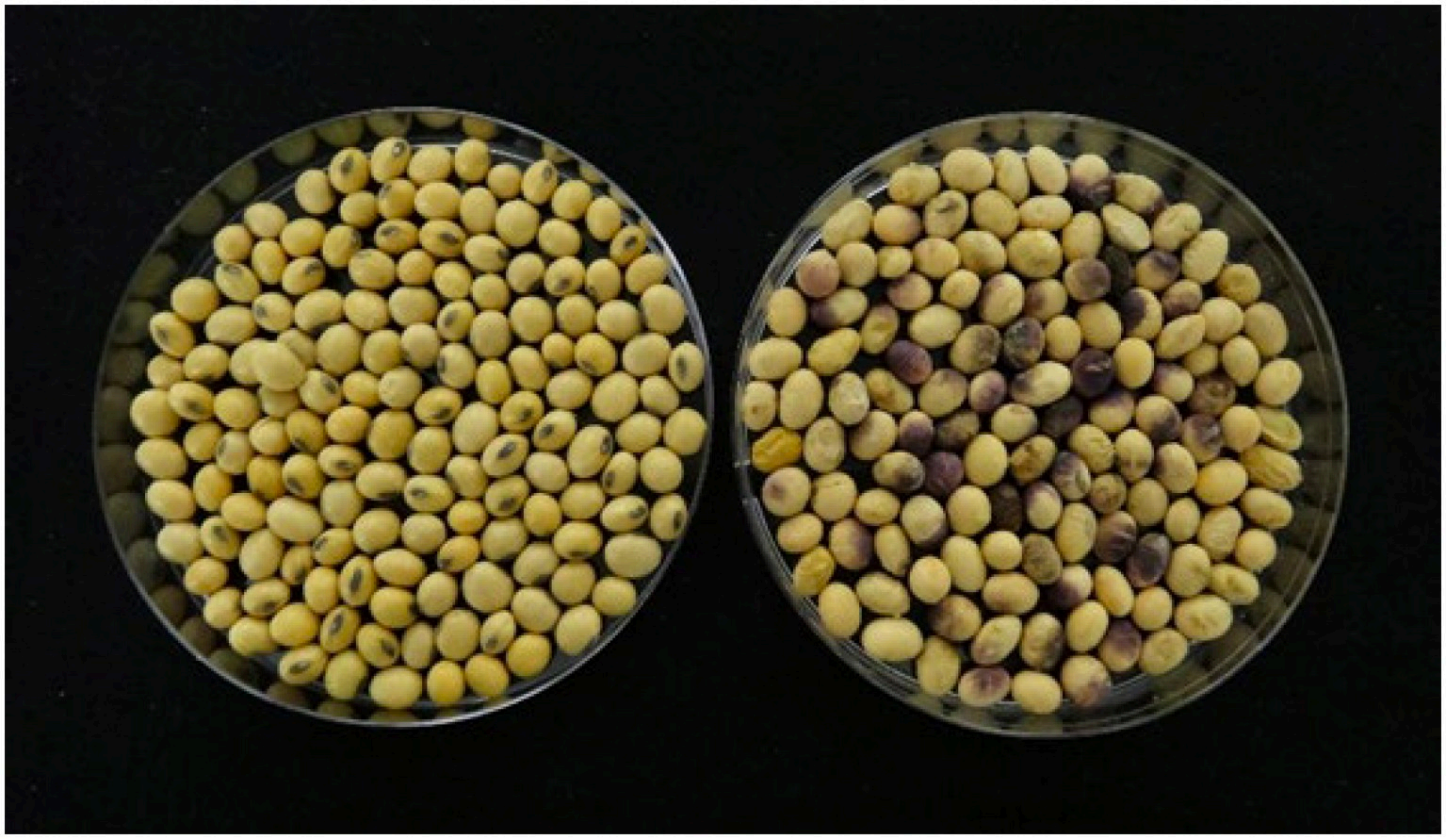
Soybean healthy seeds vs. purple seed stain. Healthy seeds without discoloration (left), while seeds with purple seed stain disease (right) usually show a pink to light or dark purple discoloration.

Purple seed stain (PSS) of soybean (*Glycine max* (L.) Merr.) is a prevalent seed disease resulting in seed decay and purple discoloration (Fig 1); reduced vigor and stand establishment [1–3], low oil content [4] and other altered composition and antioxidant properties [5]. Although significant yield loss of soybean caused by PSS in the United States has not been reported [2], it has become an increasing threat to soybean production since it can cause a significant reduction in overall seed quality and seed lot market grade, and thus undermines value of soybean worldwide [4, 6–9]. Due to the excessive rains during the harvest season in southern states in 2018, there were many issues concerning the poor quality of harvested soybean seeds which were infested by various pathogens that cause severe seed diseases including PSS (https://www.kygrains.info/).

PSS was first reported in Korea by Suzuki in 1921. The disease was described as a purple discoloration of the seeds [10]. In the United States, PSS was first reported in Indiana in 1924 [11] and then North Carolina in 1927 [12]. At present, PSS occurs in most soybean production areas worldwide [3, 7, 13].

Infected seeds usually show a pink to light or dark purple discoloration, which varies in size ranging from a small spot to covering the entire seed coat [2]. In addition to symptoms on the seed, the disease can develop on pods, stems, and leaves causing Cerospora leaf blight (CLB), an important disease associated with PSS with no correlation among cultivars for resistance [4].

Both PSS and CLB were reported to be caused by the fungal pathogen *Cercospora kikuchii* (Tak. Matsumoto & Tomoy.) M. W. Gardner [8, 11, 14, 15]. The vegetative compatibility groups and the population structure of *C*. *kikuchii* have been investigated [16, 17]. In recent studies, more *Cercospora* species have been found infecting soybean across the Americas [18]. Using a multilocus phylogenetic approach, *C*. cf. *flagellaris*. and *C*. cf. *sigesbeckiae* were reported as causal agents of PSS and CLB [18, 19].

*Cercospora* spp. produce a light-activated red perylene quinone pigment named cercosporin, which has a molecular weight of 534 and is photoactivated [20–22]. The role of cercosporin in PSS and CLB disease development has been studied. Upchurch et al. [23] developed and used *C*. *kikuchii* mutants that blocked cercosporin synthesis to demonstrate that cercosporin is important in pathogenicity. Cercosporin causes diseased tissues to develop a purplish discoloration, and it is toxic to plant cells.

Development of PSS and the growth of the causal agent are influenced by environmental factors, such as relative humidity and pH values of the substrates, as well as by light/photoperiod [24, 25], especially, during the early reproductive stages of soybean [7, 25]. The pod development stage, temperature, and pod wetness duration also affected the incidence of PSS of soybean [26].

To control PSS, several strategies have been used, such as tillage, crop rotation [27], fungicide applications at pod-filling stages [28], and the use of genetic resistance [29–34]. Planting PSS-resistant genotypes is an economical and environmentally friendly means to control the disease.

The United States Department of Agriculture (USDA) Soybean Germplasm Collection (http://www.ars-grin.gov/npgs/) has more than 19,652 accessions which originated from countries around the world, but primarily from East Asia. [35]. Some of the accessions in the USDA collection have been identified with resistance to various soybean diseases and pests [35, 36]. In previous field screenings of 208 soybean accessions in Mississippi [37], and 135 soybean lines in three southern states [38], 21 soybean accessions were reported with resistance to Phomopsis seed decay (PSD). However, information about reaction of different soybean genotypes to PSS under the natural field conditions in the Mississippi Delta area is lacking. In this study, we tested the hypothesis that soybean genotypes with different levels of resistance to PSD from the USDA Soybean Germplasm Collection could also have resistant reactions to PSS. Therefore, cross resistant genotypes to PSD and PSS could be identified. In addition, the PSS causal pathogens are common, widespread and well established in the Mississippi Delta. Hence, natural infection is feasible as an alternative option to artificial inoculation for evaluating reaction of soybean genotypes to PSS.

The objectives of this study were to evaluate the reaction of 42 diverse soybean genotypes from 15 countries in maturity groups (MGs) III, IV, and V, and to identify new sources of resistance to PSS from field trials (2010, 2011, and 2012) under the natural seed infection by *Cercospora* spp. in the Mississippi Delta of the U. S.

## Materials and methods

### Soybean lines

A total of 42 soybean genotypes were evaluated in this study that originated from 15 countries or regions in the world (Table 1). They represent maturity groups (MG) III, IV, and V including 36 plant introductions (PIs) and 6 cultivars with known reaction to PSD caused by *Phomopsis longicolla* [38, 39]. The PSS-resistant checks (PI 417361, PI 264555, and PI 407749) and susceptible checks (IA 3001, AP 350, and PI 417098) were selected based on previous tests in Arkansas [40]. Soybean cultivars SUWEON 97 and 5002T were used as cultivar checks. SUWEON97 is a cultivar originating from South Korea, while 5002T is a conventional, late group IV cultivar and a yield check in the USDA Uniform Soybean Tests-Southern States (https://data.nal.usda.gov/dataset/uniform-soybean-tests-southern-states). All soybean seed were obtained from the USDA Soybean Germplasm Collection in Urbana, IL and were increased in Costa Rica to have sufficient quantities of seed for field evaluation.

**Table 1:** Country of origin for soybean entries evaluated for reaction to natural field infection by *Cercospora* species in Stoneville, Mississippi, during 2010–2012.

Country	Number of genotypes
Argentina	1
Canada	1
China	8
France	1
India	3
Japan	6
Nepal	1
Pakistan	1
Russia	2
South Korea	4
Turkey	1
Uganda	2
Uruguay	2
USA	8
Vietnam	1
Total	42

### Field experiments

Field experiments were conducted at Stoneville, MS on a Sharkey clay soil (very-fine, smectitic, thermictic Chromic Epiaquert) in 2010, 2011 and 2012. For all experiments, seeds were planted at a rate of 33 seeds/m of row, in 2.74 m-long single-row plots with a 0.91-m between row spacing. The plot size was 0.91 m x 2.74 m. Each plot of each genotype was a single replication. Pre-emergence herbicides of Paraquat at 2.33 l/ha and metolachlor at 1.8 l/ha (Syngenta Crop Protection, Greensboro, NC) were applied on the second day after planting. After initial herbicide treatment weed removal was conducted manually. Planting dates in each year are listed in Table 2.

**Table 2:** Planting and harvest dates for evaluation of soybean for reaction to natural field infection by *Cercospora* species in Stoneville, Mississippi, in 2010, 2011, and 2012.

Planting		Harvest dates		
Year	Date	MG III	MG IV	MG V
2010	25 May	27 Aug.—7 Sep.	1–14 Sep.	14–27 Sep.
2011	20 May	1–13 Sep.	13–30 Sep.	4–5 Oct.
2012	25 April	14 Aug.—5 Sep.	21 Aug.—14 Sep.	21 Sep.—12 Oct.

The experiment was a split plot design with three MGs (MG III, MG IV, and MG V) as the main plots, arranged in a randomized complete block (RCB) design with 4 replicated blocks. The subunits consisted of 14 soybean genotypes within each MG, and the experiment was conducted for 3 years. All plants (approximately 80) in each plot were manually harvested when mature.

Plants were irrigated 2–3 times a week as needed to insure optimum plant growth and development, and natural infection was relied upon for PSS development. Irrigation was applied with overhead Rain Bird Brass Impact Sprinklers, Model #25P JDA-C (Rain Bird Co., Azusa, CA). Sprinklers were connected to a nurse wagon equipped with a Honda engine/pacer poly pump (Bell Inc., Inverness, MS).

### Seed assays

After manual harvest, 25 randomly chosen seeds (13% moisture) from each plot (100 seeds for each soybean line) were assayed to determine the percent seed infection by *Cercospora* spp., percent seed germination, and visual seed quality using the methods as previously reported [38]. Briefly, seeds were surface-disinfected in 0.5% sodium hypochlorite for 3 min, rinsed in sterile distilled water, and then placed on acidified potato dextrose agar (Difco Laboratories, Detroit, MI) adjusted to pH 4.8 with 25% (w/v) lactic acid (APDA) [37, 38]. Five seeds were placed on APDA in each 100 mm x 15 mm Petri dish where one seed was placed in the center and the others were placed equidistance around the outside of the dish, approximately 10 mm from the side with approximately 30 mm between seeds. All seed plates were incubated for four days at 24°C. *Cercospora* spp. were identified as described by Groenewald et al. [15] and Ward-Gauthier et al. [4]. Other soybean seed pathogens, such as *Alternaria* spp., *Fusarium* spp., and *Phomopsis longicolla* have different culture morphology from *Cercospora* spp. and were identified based on the description by Hartman et al. [41].

The number of seeds infected with *Cercospora* spp. was recorded and percent seed infection was calculated. Seed germination of 100 randomly selected seeds from each plot was determined using a standard soybean seed germination protocol [42]. Visual scoring of seed quality was determined using a scale of 1 to 5 as previously reported [38], in which 1 = excellent (no discolored seed); 2 = good (less than 10% discolored seed); 3 = fair (11–30% discolored seed); 4 = poor (31–50% discolored seed); and 5 = very poor (more than 50% discolored seed). Seed wrinkling, molding, mottling, and discoloration were the factors in estimating seed quality [38].

Weather data of total precipitation, number of rainy days, average maximum temperatures, and maximum relative humidity during the growing season were obtained from the Stoneville, MS weather station (http://ext.msstate.edu/anr/drec/stations.cgi?defstation=Stoneville).

### Data analyses

Statistical analysis of data was performed using the Generalized Linear Mixed procedure (PROC GLMMIX) of SAS (version 9.4, SAS Institute, Cary, NC). The model contained fixed effects for years, MG, and genotypes within each MG. Replication (Rep) within years, year interaction with MG, and year interaction with genotypes within MG were considered random effects. Since the percentage of seed infected by *Cercospora* spp. contained many zero or low values for some lines, the assumption for a normal distribution was not met. A negative binomial distribution and a log link function were used for the generalized linear mixed model. Separate analyses were performed for each MG (combined over years) and for each year of each MG. Percent seed germination, seed quality scores, and percent hard seed were calculated as the mean of each cultivar. Soybean genotypes were compared with Fisher’s least significant difference (LSD) at *P* ≤ 0.05. The PROC CORR procedure of SAS was used to compute Pearson’s correlation coefficients between percent seed infected by *Cercospora* spp. and germination, and between percent seed infection and visual seed quality.

## Results

**Fig 2:**
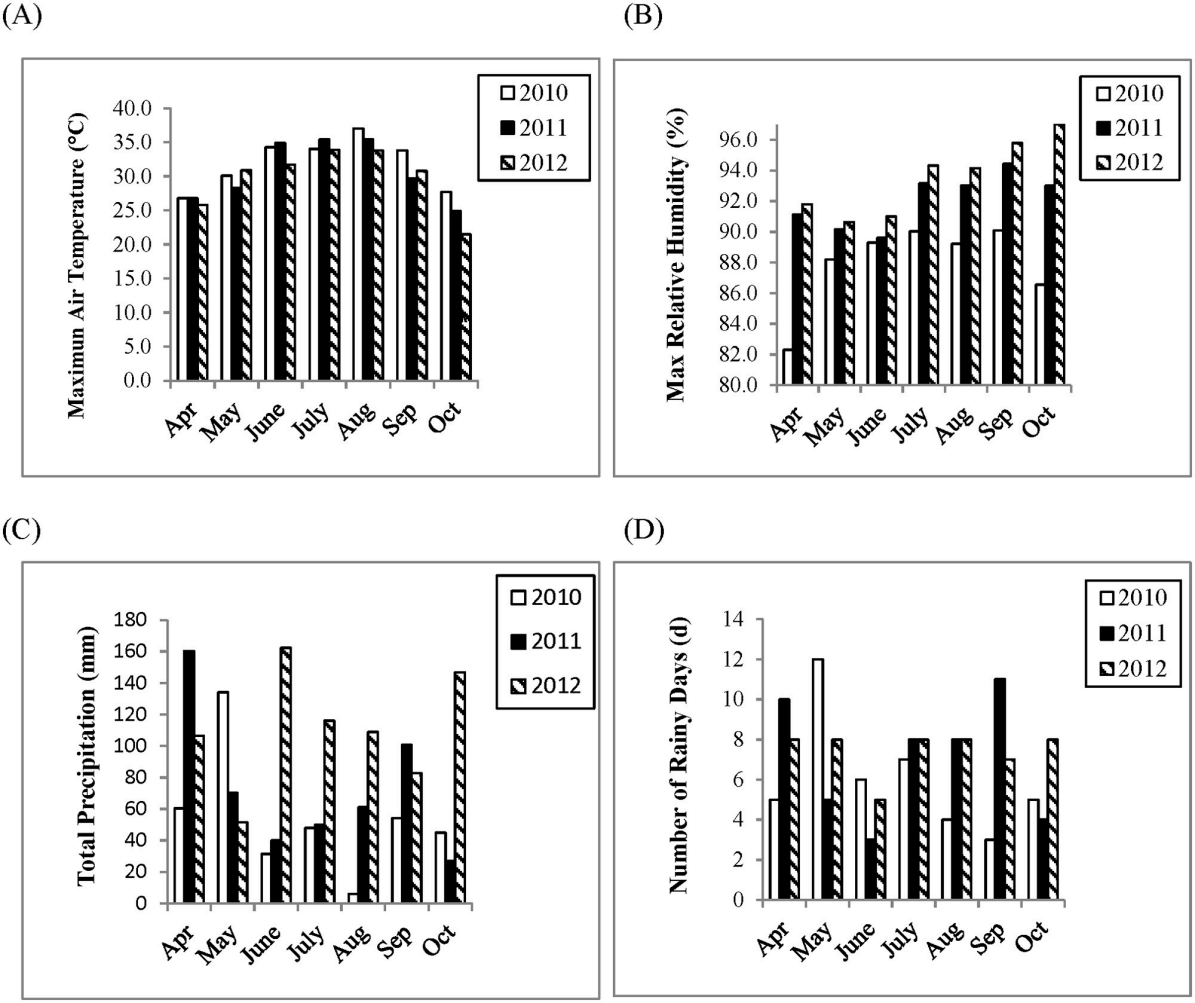
Weather information in Stoneville, MS during soybean growing seasons for the months of April through October in 2010, 2011, and 2012. **A**: Air maximum temperatures. **B**: Maximum relative humidity. **C**: Total precipitation. **D**: Number of rainy days.

The average daily maximum air temperature, relative humidity, total precipitation, and number of rainy days for each month during each growing season from 2010 to 2012 are shown in Fig 2 and S1 Table. When plants generally were at the R2 or R3 growth stages [43] in July, the amount of precipitation (116 mm) in 2012 was approximately 2.4 and 2.3 times that of 2010 (48 mm) and 2011 (50 mm), respectively. The average daily maximum air temperatures in July were 34.0°C, 35.4°C, and 33.9°C for 2010, 2011, and 2012, respectively. When plants reached R5 or R6 growth stage [43] in August, the total precipitation (109 mm) in 2012 was approximately 17.9 and 1.8 times that of 2010 (6.1 mm) and 2011 (61.2 mm), respectively. The average air temperatures in August were 37.0°C, 35.4°C, and 33.4°C for 2010, 2011, and 2012, respectively.

**Fig 3:**
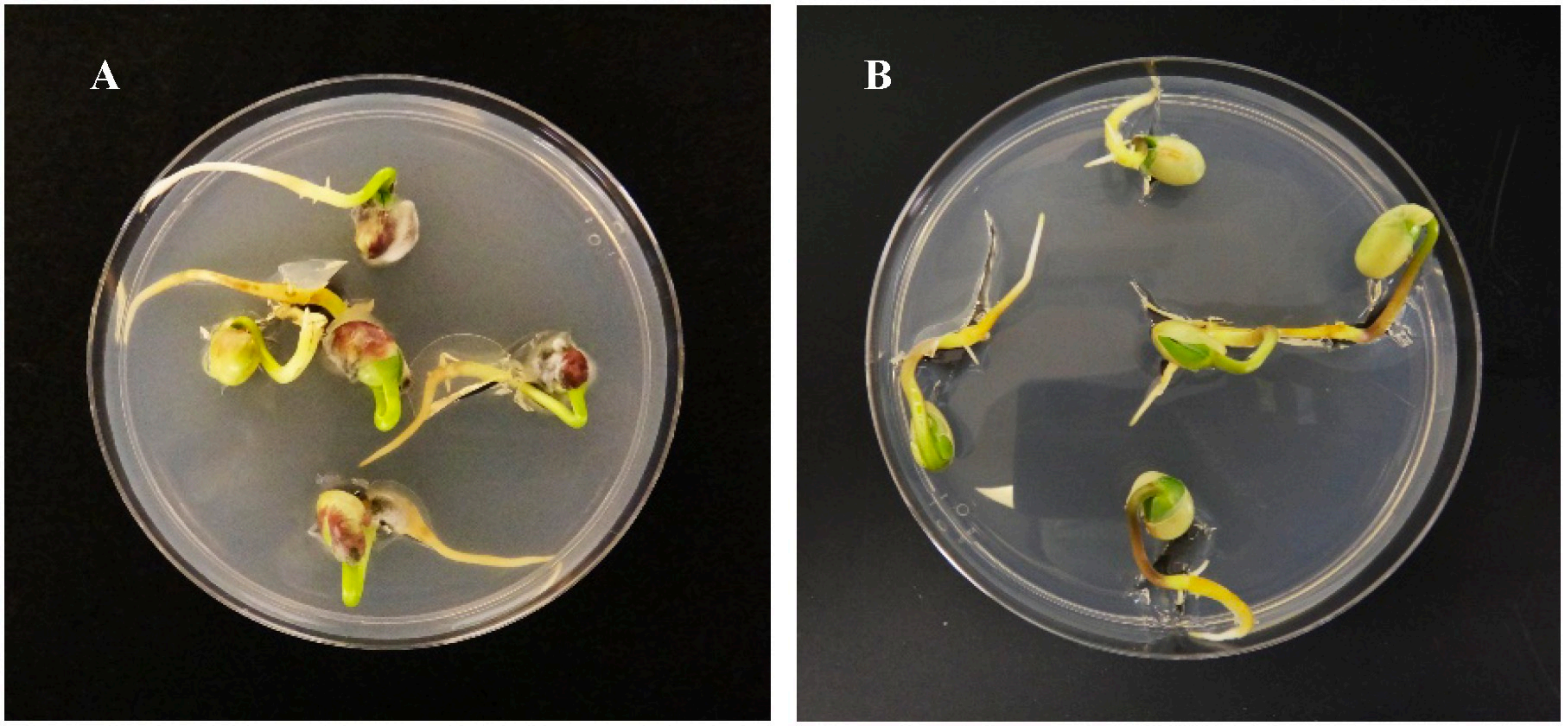
Seed plating assays on acidified potato dextrose agar (APDA). **A**. A susceptible genotype, AP 350, plated on APDA. **B**. A resistant genotype, PI 264555, plated on APDA.

Seed infection by *Cercospora* spp. as determined by the seed plating assay was severe on susceptible genotypes (Fig 3A), while reaction of resistant genotypes had no or low levels of seed infection (Fig 3B). Cultures of soybean isolates of *Cercospora* spp. on the APDA medium had the typical dense mat of mycelium with deep folds radiating from the center. Colonies of *C*. *kikuchii* were white at the edge and light grayish-olive toward the center. There was a reddish-purple pigment in the medium surrounding the colonies. The color of the pigment varied among isolates/species. Two *C*. cf. *flagellaris* isolates (obtained from this study were confirmed by multilocus phylogenetic analysis in another study [19]. The genus *Cercospora* contains over 3,000 species [15, 44]. Extensive identification of all isolates for species was not done in this study.

ANOVA of percent seed infection by *Cercospora* spp. indicated that there were significant differences (Table 3) among years (*P* = 0.0172) and genotypes (*P* ≤ 0.001). The overall means of percent *Cercospora* spp. seed infection were 7.3, 1.8, and 7.9 for 2010, 2011, and 2012, respectively. There was no significant difference between 2010 and 2012 (*P* = 0.8749). However, percent *Cercospora* spp. seed infection in 2011 was significantly lower than that in 2010 (*P* = 0.0116) and 2012 (*P* = 0.0102). Means of percent *Cercospora* spp. seed infection ranged from 0.70 to 9.67% for MG III, 2.50 to 12.33% for MG IV, and 1.50 to 9.83% for MG V (Table 4 and S2 Table). Six MG III (PI 88490, PI 504488, PI 417361, PI 548298, PI 437482, and PI 578486), seven MG IV (PI 404173, PI 346308, PI 355070, PI 416779, PI 80479, PI 346307, and PI 264555), and four MG V (PI 417567, PI 417420, PI 381659, and PI 407749) genotypes had significantly lower percent seed infection by *Cercospora* spp. than the susceptible checks and other genotypes in this study (*P ≤* 0.05). Moreover, both PI 437482 and PI 578486 (MG III) also had significantly lower percent seed infection by *Cercospora* spp. than the resistant check PI 417361. The cultivar AP350, a susceptible check for MG IV, and PI 371611 had similar high levels of seed infection by *Cercospora* spp. in all three years. The other susceptible checks, IA 3001 (MG III) and PI 417098 (MGV), had low seed infections in 2011 but their disease levels were high in 2010 and 2012 (Table 4 and S2 Table).

**Table 3:** F-test for fixed effects from analysis of variance of percentage of natural field seed infection by *Cercospora* species in Stoneville, Mississippi, in 2010, 2011, and 2012^v^.

Effect ^w^	Num DF ^x^	Den DF ^y^	*F*	*P > F*
Year	2	4	13.27	0.0172
Maturity group (MG)	2	4	1.47	0.3313
Genotype (all MGs)	39	447	4.19	< .0001
Genotype (MG III) ^z^	13	447	6.01	< .0001
Genotype (MG IV) ^z^	13	447	4.36	< .0001
Genotype (MG V) ^z^	13	447	2.23	0.0079

^v^ Analysis of variance was performed using the Generalized Linear Mixed model (PROC GLMMIX) of SAS (version 9.4, SAS Institute, Cary, NC).

^w^ Source of variance was calculated as percentage of seed infection by *Cercospora* spp. from the seed plating assays.

^x^ Numerator degree of freedom.

^y^ Denominator degree of freedom calculated based on Kenward and Roger’s approximation method [54].

^z^ Separate analysis for each maturity group combined over years.

**Table 4:** Percentage of natural field seed infection by *Cercospora* species of 42 soybean genotypes in Stoneville, Mississippi, in 2010, 2011, and 2012.

Genotype	Origin	MG ^v^	*Cercospora* spp. (%)				
			2010	2011	2012	Mean ^w^	
PI 189891	France	III	10.5	5.0	12.0	9.17	ab
PI 398697	S. Korea	III	10.5	6.5	11.0	9.33	a
PI 398752	S. Korea	III	6.0	3.0	7.0	5.33	bcd
PI 417361 ^x^	Japan	III	4.5	2.0	6.0	4.17	cd
PI 437482	Russia	III	3.5	0.0	1.0	1.50	ef
PI 504481	China	III	7.5	1.5	7.5	5.50	bcd
PI 504488	China	III	5.0	2.0	6.0	4.33	cd
PI 88490	China	III	7.0	1.0	7.0	5.00	cd
PI 416988	Japan	III	9.5	1.0	8.0	6.17	bcd
PI 547827	USA	III	11.0	2.0	14.0	9.00	abc
PI 548298	Canada	III	4.0	3.0	1.0	2.67	de
PI 578486	India	III	2.0	0.0	0.1	0.70	f
Williams 82	USA	III	16.0	1.5	9.5	9.00	abc
IA 3001 ^y^	USA	III	12.5	3.0	13.5	9.67	ab
Mean	…		7.8	2.3	7.4	5.82	
PI 158765	China	IV	16.5	3.0	17.5	12.33	a
PI 235335	Uruguay	IV	6.0	4.0	7.5	5.83	abc
PI 235346	Uruguay	IV	6.5	4.0	8.0	6.17	abc
PI 346307	India	IV	3.0	1.0	3.5	2.50	d
PI 346308	India	IV	5.0	1.0	7.0	4.33	bcd
PI 416779	Japan	IV	4.5	0.0	7.0	3.83	cd
PI 80479	Japan	IV	3.0	4.0	3.0	3.33	bcd
PI 87074	S. Korea	IV	11.0	2.0	12.0	8.33	ab
PI 264555 ^x^	Argentina	IV	3.5	0.0	4.0	2.50	d
PI 355070	USA	IV	4.0	3.0	5.0	4.00	bcd
PI 371611	Pakistan	IV	7.5	8.0	9.5	8.33	a
PI 404173	China	IV	6.0	0.0	9.0	5.00	bcd
SUWEON97 ^z^	USA	IV	8.3	2.0	14.0	8.08	ab
AP350 ^y^	USA	IV	8.0	7.0	13.5	9.50	a
Mean	…		6.6	2.8	8.6	6.01	
PI 506844	Japan	V	7.0	0.0	10.0	5.67	abc
PI 381659	Uganda	V	3.0	1.0	3.0	2.33	cd
PI 381668	Uganda	V	8.5	0.0	10.0	6.17	abc
PI 407749 ^x^	China	V	2.5	1.0	1.0	1.50	cd
PI 417567	China	V	5.5	0.0	5.0	3.50	bcd
PI 471938	Nepal	V	9.0	0.0	10.5	6.50	abc
PI 476920	Vietnam	V	4.0	2.0	4.5	3.50	abc
PI 507690	Russia	V	11.0	0.0	11.0	7.33	ab
PI 172902	Turkey	V	14.0	0.0	14.5	9.50	a
PI 407752	China	V	5.5	0.0	12.5	6.00	abc
PI 417420	Japan	V	4.0	0.0	7.0	3.67	bcd
PI 417098 ^y^	Japan	V	17.5	0.0	12.0	9.83	a
5002T ^z^	USA	V	7.5	0.0	6.5	4.67	abc
TARA	USA	V	7.0	0.0	8.0	5.00	abc
Mean	…		7.6	0.3	8.3	5.37	

^v^ Maturity group

^w^ Means followed by the same letter within a column in each MG are not significant different by the least significant difference test at *P ≤* 0.05.

^x^ Resistant check.

^y^ Susceptible check.

^z^ Cultivar check

There were differences in seed germination, visual quality, and hard seed among genotypes (Table 5 and S2 Table). The means of germination over the three years were 77.9% and 78.9% in MG III and MG IV, respectively, while MG V genotypes had a mean germination of 91.5%. For the visual quality, the mean scores ranged from 1.6 to 3.3. The resistant check, PI 417361, had the best score of 1.6 while PI 398697 had the worst score of 3.3. Percentage of hard seed ranged from 0.0% to 34.3% (Table 5 and S2 Table).

**Table 5:** Means of germination, visual quality, and hard seed of soybean genotypes in replicated field tests in Stoneville, Mississippi, during 2010–2012.

Genotype		2010			2011			2012		
	MG^r^	GM^s^	VQ^t^	HardSd^u^	GM	VQ	HardSd	GM	VQ	HardSd
PI 189891	III	72.0	3.6	13.0	51.0	2.9	23.8	84.8	2.5	2.3
PI 398697	III	94.3	2.9	1.0	82.5	3.3	32.8	94.5	1.3	0.0
PI 398752	III	60.5	2.8	18.0	88.3	2.6	11.3	96.3	1.6	1.5
PI 417361^v^	III	86.3	2.0	15.0	94.5	1.6	0.3	86.8	1.3	8.5
PI 437482	III	54.3	2.8	7.0	94.0	2.4	34.5	89.5	1.8	0.0
PI 504481	III	98.0	3.0	3.0	92.8	2.3	25.0	56.5	1.6	3.3
PI 504488	III	95.0	2.3	3.0	92.7	2.5	34.3	83.8	1.5	1.5
PI 88490	III	86.8	2.9	4.0	86.3	1.8	0.0	78.7	3.2	2.3
PI 416988	III	92.5	1.9	4.0	82.5	2.0	6.0	74.8	1.8	2.3
PI 547827	III	72.3	3.3	7.0	92.0	2.5	34.3	43.3	2.0	0.0
PI 548298	III	55.5	3.1	5.0	75.8	2.9	17.3	78.3	3.4	0.0
PI 578486	III	34.0	3.6	0.0	59.0	2.4	14.8	87.8	2.3	4.5
Williams 82	III	60.3	3.1	5.0	75.3	2.5	20.3	78.0	3.6	0.0
IA 3001^x^	III	59.5	2.6	0.0	78.8	3.0	27.0	72.3	1.9	2.5
Mean	…	72.9	2.8	6.1	81.8	2.5	20.1	78.9	2.1	2.0
LSD^y^	…	11.0	0.4	5.1	13.4	0.5	9.4	6.5	0.5	3.7
PI 158765	IV	95.8	2.9	1.0	91.0	2.4	18.7	87.5	3.3	0.3
PI 235335	IV	74.3	2.9	11.0	65.3	2.5	18.5	76.5	1.6	7.5
PI 235346	IV	86.0	2.6	1.0	74.3	1.9	13.5	79.3	2.6	6.0
PI 346307	IV	64.3	3.3	10.0	76.3	2.3	8.5	72.3	2.8	2.0
PI 346308	IV	80.3	3.9	4.0	90.3	1.9	6.5	84.3	2.6	7.8
PI 416779	IV	55.8	2.9	5.0	61.7	2.0	20.0	69.5	2.8	26.8
PI 80479	IV	82.5	4.0	0.0	66.3	1.6	15.8	64.0	3.3	0.3
PI 87074	IV	94.0	3.3	0.0	92.8	2.5	13.0	72.0	2.8	0.5
PI 264555^v^	IV	84.5	2.0	1.0	92.0	2.0	4.3	80.0	9.1	3.0
PI 355070	IV	77.5	3.4	0.0	82.0	2.5	18.3	84.5	2.4	1.5
PI 371611	IV	85.3	2.8	3.0	60.5	2.9	12.5	74.3	3.1	0.8
PI 404173	IV	96.3	2.6	0.0	82.3	2.5	26.3	86.3	2.4	1.0
SUWEON97^z^	IV	81.0	2.9	1.0	78.0	2.0	15.8	78.0	3.6	3.8
AP350 ^x^	IV	80.5	2.9	0.0	73.0	2.9	12.0	81.8	2.5	1.5
Mean	…	81.3	3.0	2.6	77.5	2.3	14.5	77.9	3.2	4.2
LSD	…	16.9	0.6	7.2	14.7	1.3	13.5	18.1	0.7	4.7
PI 506844	V	99.3	2.4	0.0	86.5	2.9	9.3	83.0	2.9	1.0
PI 381659	V	96.8	2.5	0.0	95.0	1.9	2.3	85.0	2.6	0.3
PI 381668	V	100.0	2.6	0.0	96.8	2.4	0.8	90.8	2.6	0.3
PI 407749^v^	V	82.8	2.6	6.0	96.3	2.0	0.8	84.5	2.9	2.0
PI 417567	V	96.5	2.3	2.0	96.8	1.8	2.0	95.3	2.1	0.0
PI 471938	V	99.5	2.0	0.0	96.8	2.4	1.0	93.0	2.1	0.5
PI 476920	V	99.5	2.4	1.0	91.5	1.9	5.5	85.3	2.3	4.0
PI 507690	V	97.8	2.8	0.0	97.8	1.8	0.3	70.8	3.4	0.0
PI 172902	V	95.5	2.4	6.0	98.5	2.1	0.0	83.5	2.6	0.3
PI 407752	V	92.8	2.0	1.0	70.3	1.5	24.5	81.5	2.3	3.5
PI 417420	V	97.0	2.3	2.0	97.5	2.0	1.5	88.0	2.5	0.0
PI 417098 ^x^	V	98.3	2.8	0.0	97.3	2.1	0.3	78.8	3.1	0.0
5002T ^z^	V	95.0	2.4	2.0	93.3	1.8	2.8	93.5	2.3	0.0
TARA	V	95.3	2.1	5.0	97.8	2.4	0.3	74.8	3.3	1.0
Mean	…	96.1	2.4	1.8	93.7	2.1	3.6	84.8	2.6	0.9
LSD	…	3.3	0.6	5.5	7.3	0.6	5.6	18.2	0.3	3.1

^r^ MG = maturity group.

^s^ GM = percentage of seed germination.

^t^ VQ = visual quality of seeds from non-inoculated tests. Seeds were assessed using a scale of 1 to 5 where 1 = excellent (no bad seed); 2 = good (less than 10% bad seed); 3 = fair (11–30% bad seed); 4 = poor (31–50% bad seed); and 5 = very poor (more than 50% bad seed). Factors considered in estimating seed quality were: development of seed wrinkling, molding, mottling, and discoloration.

^u^ HardSd = percentage of hard seed.

^v^ Resistant check.

^x^ Susceptible check.

^y^ LSD = Fisher’s protected lease significant difference (LSD) test (*P* ≤ 0.05) for means within the column in each maturity group.

^z^ Cultivar check.

The PROC CORR analyses indicated that there was a significant negative correlation between percent seed infected by *Cercospora* spp. and germination (r = -0.1170, *P ≤* 0.0089) and between percent seed infection and percent hard seed (r = -0.1655, *P ≤* 0.0020). However, percent seed infection was positively correlated with the score of visual seed quality (r = 0.1314, *P ≤* 0.0033), (Table 6). In addition, germination significantly and negatively correlated with the score of visual seed quality (r = -0.2489, *P ≤* 0.0001) and the percentage of hard seed (r = -0.1990, *P ≤* 0.0001). Correlation between visual seed quality score and percentage of hard seed was not significant (Table 6).

**Table 6:** Pearson correlation coefficients between percentage of seed infection with *Cercospora* species, germination, and seed visual quality in replicated field tests in Stoneville, Mississippi, in 2010, 2011, and 2012.

	***Cercospora***^u^	**Germination**^v^	**Visual quality**^w^	**Hard Seed**^x^
***Cercospora***	1.0000	-0.1170 ^y^	0.1314	-0.1655
		(0.0089) ^z^	(0.0033)	(0.0002)
**Germination**		1.0000	-0.2489	-0.1990
			(<0.0001)	(<0.0001)
**Visual quality**			1.0000	0.0380
				(0.3968)
**Hard Seed**				1.0000

^u^ Percentage of seed infection by *Cercospora* spp. that cause purple seed stain on soybean.

^v^ Percentage of seed germination.

^w^ Seeds were assessed using a scale of 1 to 5 where 1 = excellent (no bad seed); 2 = good (less than 10% bad seed); 3 = fair (11–30% bad seed); 4 = poor (31–50% bad seed); and 5 = very poor (more than 50% bad seed). Factors considered in estimating seed quality were: development of seed wrinkling, molding, mottling, and discoloration.

^x^ Percentage of hard seed.

^y^ Pearson correlation coefficients.

^z^ Probability.

## Discussion

Experiments were designed to evaluate 42 diverse maturity group III, IV, and V soybean genotypes for their reaction to natural field infection by *Cercospora* spp. in 2010, 2011, and 2012. Significant differences in percent *Cercospora* seed infection among genotypes enabled identification of resistant genotypes to PSS in all three maturity groups.

Previous research reported a greater percentage of *C*. *kikuchii* seed infection in MG III and MG IV than MG V soybean genotypes [40]. It is likely due to the early soybean production system in Arkansas, where MG III and MG IV soybean cultivars are planted in late April to early May. The early planting could result in plants developing and maturing under conditions more favorable for seed infection by *Cercospora* spp., *P*. *longicolla*, and other fungal pathogens [30, 45]. In this study, no significant MG interaction was observed although there were significant differences in the percent *Cercospora* seed infection among genotypes within each MG. The soybean genotypes were planted on 25 May in 2010, 20 May in 2011, and 25 April in 2012, respectively. The effect of planting dates on *Cercospora* seed infection in the same year or different years in the Mississippi Delta region remains in need of further study.

The year to year interaction from the ANOVA analysis indicated that seed infection by *Cercospora* spp. was affected by yearly environmental differences. In this study, seed infection in 2010 and 2012 was higher than that in 2011. Roy and Abney [7] conducted experiments with inoculation at R2 (full bloom), R3, R5, R6 (intermediate stages of pod and seed development), and R8 (95% of pods are brown at maturity) and demonstrated that seed infection was favored most by inoculation at R2 and R3. They found that the weather at the time of inoculation and several days afterward were critical for PSS development. Temperature was considered an influencing factor affecting survival and penetration of *C*. *kikuchii* [7]. In addition, Jones [46] found that PSS was incited by several *Cercospora* species when they were inoculated into attached developing pods based on his mycelial injection/inoculation studies, inferring that PSS development required the presence of the pathogen in the soybean pods.

In view of the weather conditions in this study when soybean plants reached R2 to R3 growth stages in July, the total precipitation in 2012 was more than 2 times higher than that in 2010 and 2011. However, there was no significant difference of percent *Cercospora* spp. seed infection between 2010 and 2012. Percent *Cercospora* spp. seed infection in 2011 was significantly lower than that in 2010 and 2012. Average relative humidity for July was similar for all three years. In July of 2012, the relative humidity averaged 94.3%, and was slightly lower at 90% in 2010 and 93.2% in 2011.

The weather conditions during 2010 and 2011 were very similar, but percent seed infection by *Cercospora* spp. in 2011 was much lower than that in 2010. It appears that general environmental conditions could not explain this difference. Cai et al. [47] found a new lineage of *C*. *kikuchii* with lower virulence that dominated the population compared to the fewer than 5% of isolates clustered with the old lineage in Louisiana. Although it was not addressed in this study, variability of isolate virulence and differences in the pathogen population could possibly explain differences in seed infection in these two years.

*Cercospora* is a large genus with more than 3,000 described species [15, 44], containing some of the most economically important plant pathogens. Since 1921, when the disease was first discovered, *C*. *kikuchii* was considered the single pathogen causing PSS on soybean. However, other *Cercospora* species such as *C*. cf. *flagellaris* and *C*. cf. *sigesbeckiae*, recently have been reported to infect soybean as causal agents of PSS and CLB [18–19]. The population of *Cercospora* species causing PSS on soybean in Mississippi Delta has not been determined. Experiments are underway to characterize isolates of *Cercospora* species collected from this study using morphological and molecular approaches.

Seed germination is one of the important components of soybean seed quality. Germination involves the physiological processes following seed water imbibition and rehydration that begins embryo development and ends upon protrusion of the embryonic axis (radicle) through the seed coat [48]. The effect of *C*. *kikuchii* on seed germination has been controversial. Some studies showed no effect on seed germination [49, 50], whereas others found reduced seed germination from *C*. *kikuchii* infection [6, 9]. Results from the tests of 13 cultivars with four isolates [1] showed that the effect of *C*. *kikuchii* on seed germination differed among isolates and cultivars. This may explain, in part, the different results by Han [49], Lehman [50] and others [6, 9].

In our study, although there was a significant negative correlation between percent seed infected by *Cercospora* spp. and germination (*P ≤* 0.0089), the correlation efficient was only– 0.1170. Some genotypes had low levels of *Cercospora* seed infection, but also had low seed germination, while some genotypes had high levels of seed infection, but had high percentages of seed germination. For example, in 2010, PSS-resistant line PI 578486 had 2% *Cercospora* seed infection, but only 34% germination while a PSS-susceptible line PI 507690 had 11% *Cercospora* seed infection, but 97.8% seed germination in 2010. Although other seed pathogens might be involved, different genotypes of soybean had different reactions to the same population of *Cercospora* spp. in the same year and same location under similar environmental conditions. Therefore, genetic make-up of soybean genotype likely influenced resistance or susceptibility to PSS.

Another issue is related to the antagonism between *Cercospora* spp. and other seed-borne fungal pathogens of soybean. Many seed-borne pathogens have been reported causing soybean diseases [41]. The interaction between *C*. *kikuchii* and fungi of the *Diaporthe/Phomopsis* complex has been reported [51, 52]. Roy and Abney [52] found that soybean seed infection by *D*. *phaseolorum* var. *sojae* and var. *caulivora* was significantly reduced in soybean plants that were inoculated with *C*. *kikuchii* as compared to non-inoculated plants, indicating that *C*. *kikuchii* was an antagonist of certain natural-occurring fungi on soybean seeds, especially *D*. *phaseolorum* var. *sojae*. Therefore, research involving soybean plants inoculated with *C*. *kikuchii*, the occurrence and frequency of other seed-borne pathogens should be taken into account [52].

Both PSS and PSD are important soybean diseases affecting seed quality [3, 53]. Of 17 lines with different levels of resistance to PSS identified from this study, nine soybean genotypes (PI 417361, PI 504488, PI 88490, PI 346308, PI 416779, PI 417567, PI 381659, PI 417567, and PI 407749) were the previously reported lines with resistance to Phomopsis seed decay of soybean [38]. Among them, PI 417361 was also identified as a resistant line to PSS from the field tests in Arkansas in 2007 and 2008 [40]. All of these soybean lines could be useful in breeding programs for developing resistant soybean to both seed diseases.

## Supporting information

S1 Table Weather information in Stoneville, Mississippi, in 2010, 2011, and 2012. **A**: Air maximum temperatures. **B**: Maximum relative humidity. **C**: Total precipitation. **D**: Number of rainy days.
(XLSX)

S2 Table Seed evaluation of soybean genotypes harvested in Stoneville, Mississippi, in 2010, 2011, and 2012. (CSV)
